# Key elements involved in Epstein–Barr virus-associated gastric cancer and their network regulation

**DOI:** 10.1186/s12935-018-0637-5

**Published:** 2018-09-21

**Authors:** Jing-jing Jing, Ze-yang Wang, Hao Li, Li-ping Sun, Yuan Yuan

**Affiliations:** Tumor Etiology and Screening Department of Cancer Institute and General Surgery, The First Hospital of China Medical University, Key Laboratory of Cancer Etiology and Prevention (China Medical University), Liaoning Provincial Education Department, Shenyang, Liaoning China

**Keywords:** Transcription factor, Noncoding RNA, Gene regulatory network, Bioinformatics, EBV-associated gastric cancer

## Abstract

**Background:**

The molecular mechanism of Epstein–Barr virus (EBV)-associated gastric cancer (EBVaGC) remains elusive. A collection of molecular regulators including transcription factor and noncoding RNA (ncRNAs) may affect the carcinogenesis of EBVaGC by regulating the expression and function of key genes. In this study, integration of multi-level expression data and bioinformatics approach was used to identify key elements and their interactions involved in mechanism of EBVaGC and their network regulation.

**Methods:**

Data of the gene expression profiling data sets (GSE51575) was downloaded from GEO database. Differentially expressed genes between EBVaGC and normal samples were identified by GEO2R. Gene ontology and pathway enrichment analyses were performed using R packages Cluster profiler. STRING database was used to find interacting proteins between different genes. Transcription factors in differentially expressed genes were obtained from TF Checkpoint database. Using Cytoscape, we built transcription factor regulation network. miRNAs involved in the gene-interacting proteins and the miRNA-targeted lncRNA were predicted through miRWalk. Using ViRBase, EBV related miRNA regulation network was built. Overlapping genes and regulators of the above three networks were further identified, and the cross network was constructed using Cytoscape software. Moreover, the differential expressions of the target genes and transcription factors in the cross network were explored in different molecular subtypes of GC using cBioPortal. By histological verification, the expression of two main target genes in the cross network were further analyzed.

**Results:**

A total of 104 genes showed differential expressions between EBVaGC and normal tissues, which were associated with digestion, G-protein coupled receptor binding, gastric acid secretion, etc. Pathway analysis showed that the differentially expressed genes were mainly enriched in gastric acid secretion and protein digestion and absorption. Using STRING dataset, a total of 54 proteins interacted with each other. Based on the transcription factor network, the hub transcription factors IRX3, NKX6-2, PTGER3 and SMAD5 were identified to regulate their target genes SST and GDF5, etc. After screening and matching in miRwalk datasets, a ceRNA network was established, in which the top five miRNAs were hsa-miR-4446-3p, hsa-miR-5787, hsa-miR-1915-3p, hsa-miR-335-3p and hsa-miR-6877-3p, and the top two lncRNAs were RP5-1039K5.19 and TP73-AS1. According to the EBV related miRNA regulation network, CXCL10 and SMAD5 were found to be regulated by EBV-miR-BART1-3p and EBV-mir-BART22, respectively. By overlapping the three networks, CXCL10, GDF5, PTGER3, SMAD5, miR-6877-3p, RP5-1039K5.19, TP73-AS1, EBV-miR-BART1-3p and EBV-mir-BART22 were found to be key elements of regulation mechanism of EBVaGC. CXCL10, GDF5, PTGER3 and SMAD5 were also differentially expressed among the four molecular subtypes of GC. The histological verification experiment showed differential expressions of the two main target genes GDF5 and CXCL10 between EBVaGC and non-tumor tissues as well as EBVnGC.

**Conclusion:**

In the current study, our results revealed key elements and their interactions involved in EBVaGC. Some hub transcription factors, miRNAs, lncRNAs and EBV related miRNAs were observed to regulate their target genes. Overlapping genes and regulators were observed in diverse regulation networks, such as CXCL10, GDF5, PTGER3, SMAD5, miR-6877-3p, RP5-1039K5.19, TP73-AS1, EBV-miR-BART1-3p and EBV-mir-BART22. Moreover, CXCL10, GDF5, PTGER3 and SMAD5 were also differentially expressed among the four molecular subtypes of GC. The histological verification experiment showed differential expressions of the two main target genes GDF5 and CXCL10 between EBVaGC and non-tumor tissues as well as EBVnGC. Therefore, the identified key elements and their network regulation may be specifically involved in EBVaGC mechanisms.

**Electronic supplementary material:**

The online version of this article (10.1186/s12935-018-0637-5) contains supplementary material, which is available to authorized users.

## Background

Gastric cancer (GC) is the fourth most common cancer in the world, ranking second in the causes of cancer death [1]. It is a complex disease with great heterogeneity that can be divided into four molecular groups based on genomic characteristics and clinical features, including chromosomal instability (CIN), genomically stable (GS), microsatellite instability (MSI) and EBV-associated GC (EBVaGC) [2]. EBV is detected in GC cells rather than in noncancerous gastric mucosa, and shows a clonal nature in neoplastic cells. It is therefore considered to have a causal role in GC [3, 4]. Molecular characterization of EBVaGC has been described recently [2]. However, the pathogenic mechanism of EBVaGC remains elusive.

Gene misregulation plays a critical role in tumorigenesis and progression [5]. Regulation of gene expression includes a great variety of mechanisms that increase or decrease the specific gene products. Gene regulatory network is a collection of molecular regulators that interact with each other to govern the gene expression and function, which has been getting increasing attention for facilitation of gaining insight into the transcriptional and epigenetic regulation patterns in cancers [6, 7]. At the transcriptional level, transcription factors (TFs) are the main regulators. They can bind to the DNA regions of enhancer or promoter adjacent to the target genes that they regulate [8, 9]. Noncoding RNAs (ncRNAs) have been shown to regulate gene expression serving as an important type of epigenetic regulation mechanism [10, 11]. Two of the main types of ncRNAs, which are microRNAs (miRNAs) and long ncRNAs (lncRNAs), can suppress each other as competing endogenous RNAs (ceRNAs) and form a regulatory ceRNA network (lncRNAs–miRNAs–mRNAs) to regulate target mRNAs [12]. In addition, not only mammals but also viruses encode miRNAs. EBV was the first virus in which viral miRNAs were found. Recently, it has been commonly accepted that EBV also encodes for plenty of miRNAs, such as BART cluster and BHRF cluster [13, 14]. These miRNAs were observed to promote viral latency or cancer development by targeting both viral and cellular genes [15–17].

Given the importance of TFs and ncRNAs, it is of great interest to construct gene regulatory networks based on TFs and ncRNAs for exploring the biological processes of EBVaGC. With the increasing availability of multi-level expression data from cancer and normal tissues, new opportunities for the extraction and integration of large data sets such as gene expression omnibus (GEO) may help to provide a more comprehensive understanding of cancer [18, 19]. In this study, we integrated expression data to identify differentially expressed mRNAs and the corresponding TFs, miRNAs, and lncRNAs involved in EBVaGC. Regulatory networks including TF–mRNA, lncRNA–miRNA–mRNA, EBV encoded miRNA–mRNA and their overlap were analyzed, which possibly provide a new avenue for investigating the regulation mechanisms of EBVaGC.

## Materials and methods

### Microarray data

GSE51575 is an mRNA profiling for EBVaGC. By downloading the GSE51575 microarray data, the adjacent normal tissues from 26 gastric cancer patients were used as control to be compared with 12 EBVaGC tissues.

### Data processing

As an interactive online tool, GEO2R (http://www.ncbi.nlm.nih.gov/geo/geo2r/) can be used to compare two or more sets of samples to determine differentially expressed genes in the GEO series [20]. In order to ensure the accuracy of the results, we used GEO2R to filter differentially expressed genes between EBVaGC and normal samples separately in each of the data sets. FDR < 0.05 and |logFC| > 4 were considered statistically significant. Duplicate gene probes and unspecific probes will be removed.

### Gene ontology and Kyoto encyclopedia of genes and genomes (KEGG) pathway enrichment analyses

Gene ontology analysis (GO) is a major bioinformatics tool to unify the representation of genes and gene products [21]. It contains three categories of terms including cellular component, molecular function, and biological process. KEGG is a set of databases containing information about genomes, biological pathways, diseases and chemicals [22]. GO and KEGG pathway enrichment analyses were performed using R packages Cluster profiler. P < 0.05 was considered statistically significant.

### Construction of transcription factor regulation network

STRING database was used to find interacting proteins between different genes [23]. Cytoscape software was used to screen for the hub protein. TF Checkpoint database was used to find the TFs in differentially expressed genes. The TFs in the PPI network were considered as the hub TFs. Using Cytoscape [24], we built transcription factor regulation network.

### Construction of ceRNA regulatory network

miRWalk is a database that can predict miRNA target genes [25]. We conducted a systematic analysis on the interaction between significantly modulated miRNAs and mRNAs considering an inverse expression correlation using MiRwalk. We ordered miRNAs on the basis of the connection numbers of target genes to select the top five miRNAs as hub miRNA. We then predicted the miRNA–targeted lncRNA in similar ways through miRWalk. The top 2 lncRNAs were selected as the hub lncRNA. Cytoscape software was used to construct ceRNA interaction network. These selected hub miRNAs and lncRNAs indicated that they can regulate more differentially expressed genes.

### Construction of EBV related miRNA regulation network

ViRBase (http://www.rna-society.org/virbase) is an online tool that can predict virus-host ncRNA-associated interactions [26]. Using ViRBase, we predicted the EBV related miRNA. Then we built the EBV related miRNA regulation network by Cytoscape.

### Construction of cross network

Overlapping genes and regulators of the above three networks were further identified, and the cross network was constructed using Cytoscape software.

### Exploring differential expressions of the target genes and transcription factors in the cross network in different molecular subtypes of GC using cBioPortal

Using cBioPortal (http://cbioportal.org), a web resource for exploring, visualizing, and analyzing multidimensional cancer genomics data [27], we analyzed the mRNA expressions of the target genes and TFs in the cross network in four different subtypes of GC (EBVaGC, GS–GC, MSI–GC, CIN–GC). P < 0.05 was considered to be statistical significant.

### Verification experiment of the target genes in the cross network using human tissues

**Fig. 1:**
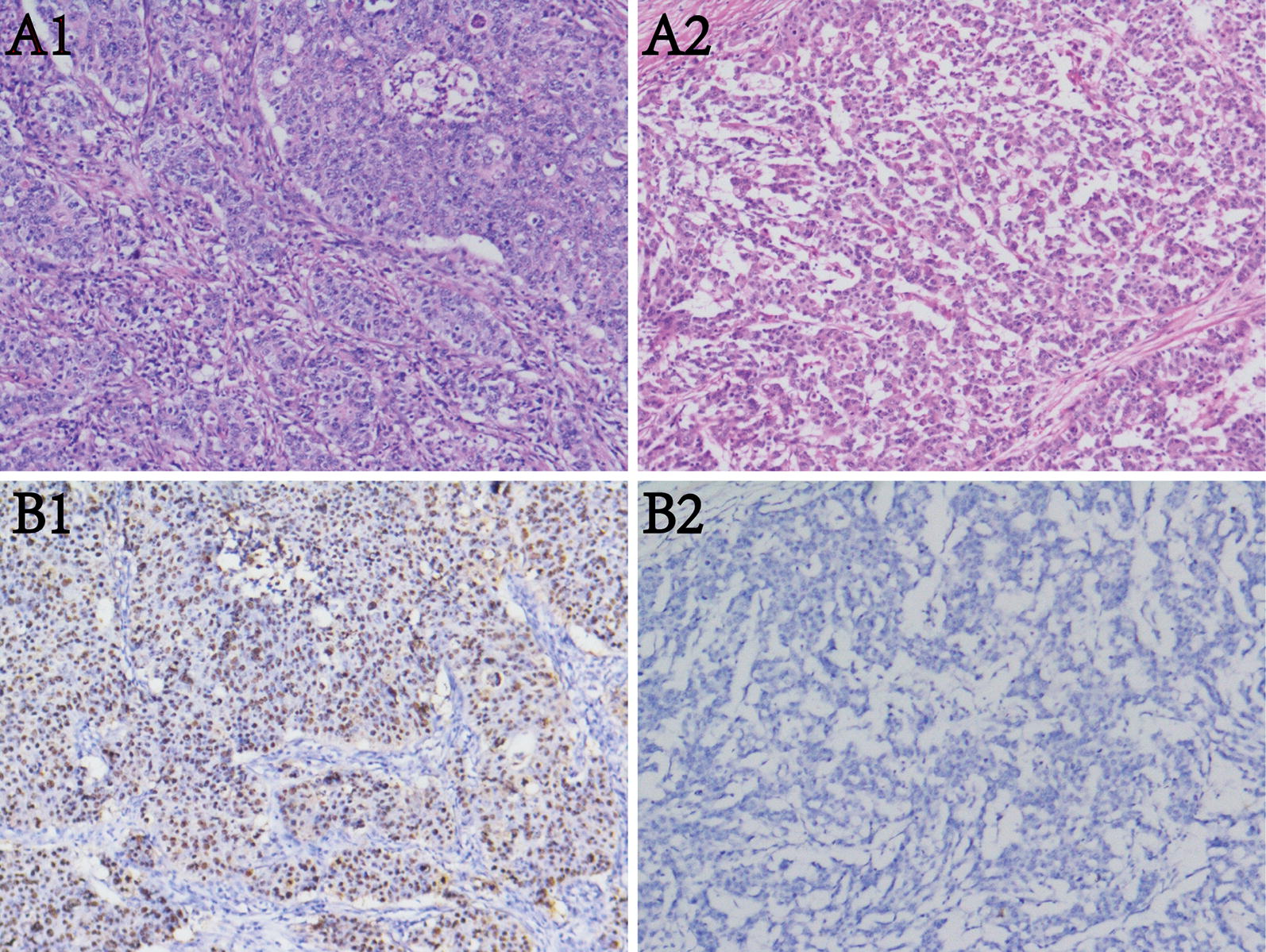
Detection of Epstein–Barr virus encoded small RNAs (EBERs) by in situ hybridization in gastric cancer tissues. A1&A2: H&E staining of EBVaGC and EBVnGC. B1&B2: EBER positive and negative staining in the nuclei of tumor cells (original magnification × 100)

Further, using 10 pairs of tumor tissues (5 EBVaGC and 5 EBVnGC) and adjacent non-tumor tissues, we detected the mRNA expression of two main target genes GDF5 and CXCL10 (approved by the Human Ethics Review Committee of the First Hospital of China Medical University). EBV was identified by the expression of EBV-encoded small RNA (EBER). In situ hybridization (ISH) with a complementary digoxigenin-labeled oligomer was used to detect the EBER according to the manufacturer’s instructions (EBER Detection Kit, ZSGB-BIO). The hybridization signal was detected by diaminobenzidine (DAB) and positive nuclear signal was recognized as dark brown nuclear staining under light microscopy (Fig. 1). Sections from a patient with known EBER-positive GC were used for a positive control. Quantitative real-time PCR was used to detect the mRNA expression. The primers were listed in Additional file 1: Table S1. P < 0.05 was considered to indicate significant differences.

## Results

### Identification of differentially expressed genes in EBVaGC

**Fig. 2:**
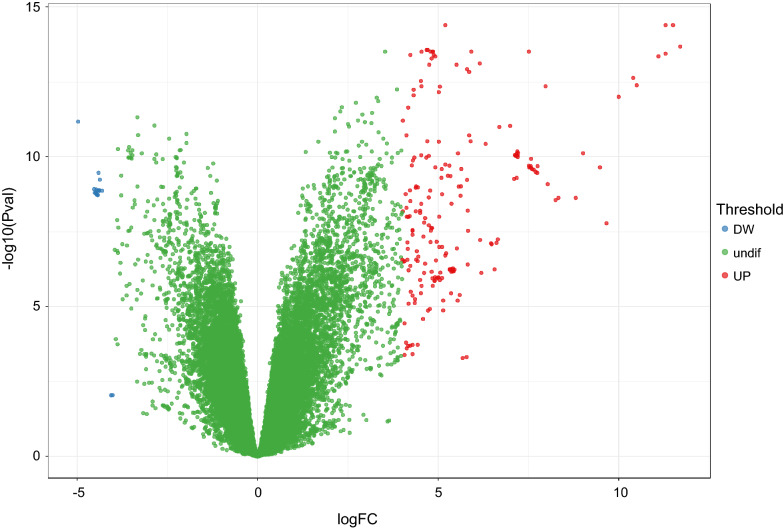
Volcano plot of the differentially expressed genes in gene expression dataset GSE51575. Red color is indicative of up-regulated and green color of down-regulated genes in normal controls compared to EBVaGC. Blue color indicates genes that they are not differentially expressed in statistical significant manner (the cutoff values of FDR < 0.05 and |logFC| > 4)

According to differential expression analysis, a total of 216 gene probes showed differences, among which 199 genes were low expressed in EBVaGC and 17 genes were highly expressed in EBVaGC (Fig. 2). After removing the duplicate gene probes and unspecific probes, 100 low expressed genes and 4 highly expressed genes were remained. The identified differentially expressed genes were listed in Additional file 1: Table S2.

### GO and KEGG pathway functional enrichment analyses

**Fig. 3:**
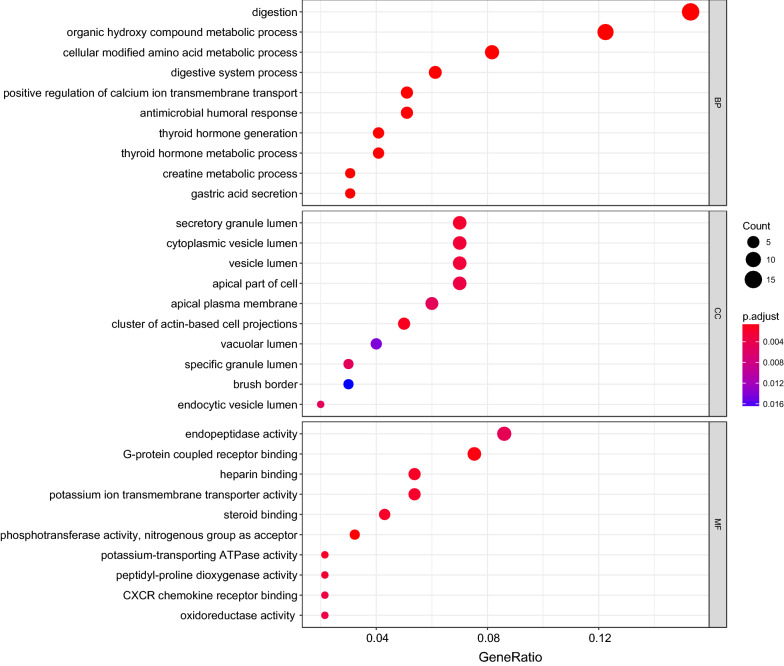
Bubbleplot for GO enrichment of differentially expressed genes. The gene ratio is assigned to the x-axis and the description of pathway to the y-axis. The area of the displayed circles is proportional to the number of genes assigned to the term and the color corresponds to the adjusted *P* value

As shown in Fig. 3, differentially expressed genes were mainly associated with digestion, G-protein coupled receptor binding, gastric acid secretion and so on. Differential genes were mainly located in cytoplasmic vesicle lumen. By KEGG enrichment analysis, the differential genes were mainly associated with the gastric acid secretion and protein digestion and absorption (Table 1).

**Table 1:** KEGG enrichment analysis for the differential expressed genes

ID	Description	Gene ratio	Bg ratio	P value	P adjust	q value	Gene ID	Count
hsa04971	Gastric acid secretion	6/54	75/7383	1.58E−05	0.0012128	0.0010942	495/496/6750/3773/9992/887	6
hsa04974	Protein digestion and absorption	6/54	90/7383	4.48E−05	0.0017241	0.0015556	643834/1280/1358/6564/10136/23436	6

### Transcription factor regulation network

**Fig. 4:**
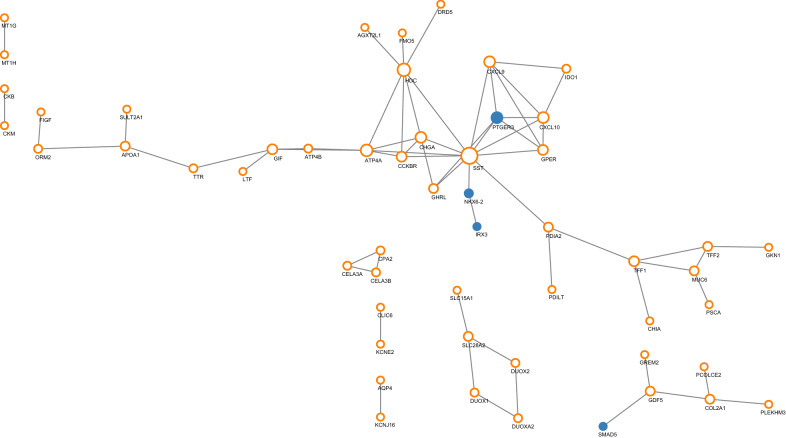
Transcription factor regulatory network (orange circles indicate common genes, blue dots indicate transcription factors, and size increases with degree)

We then built the transcription factor regulation network based on differential expressed 104 genes. Using STRING dataset, a total of 54 proteins interacted with each other. Using CytoScape, the hub transcription factors were IRX3, NKX6-2, PTGER3, and SMAD5, targeting SST and GDF5 separately. As shown in Fig. 4, orange circles indicate common genes, blue dots indicate transcription factors, and size increases with degree. Compared with normal tissues, the expression levels of the four hub transcription factors IRX3, NKX6-2, PTGER3, and SMAD5 were down-regulated, with the logFC value of − 4.39, − 5.83, − 4.18 and − 4.64, separately.

### ceRNA regulation network

**Fig. 5:**
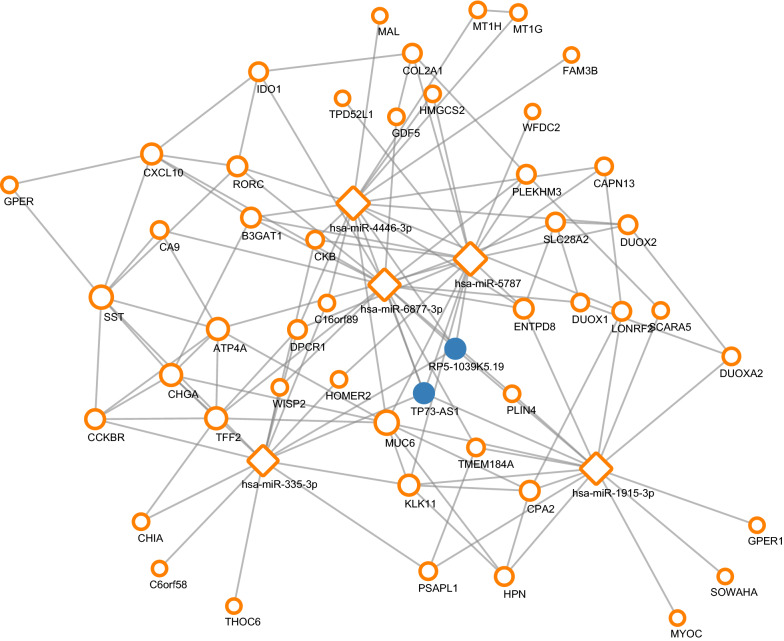
ceRNA regulatory network (orange circles indicate target genes, orange squares indicate miRNAs, blue dots indicate lncRNAs, and size increases with degree)

After screening and matching in miRwalk datasets, an integrated lncRNA–miRNA–mRNA network was established. The top five miRNAs were hsa-miR-4446-3p, hsa-miR-5787, hsa-miR-1915-3p, hsa-miR-335-3p and hsa-miR-6877-3p. A total of 47 genes were regulated by hub miRNAs. The top two lncRNAs were RP5-1039K5.19 and TP73-AS1 (Fig. 5).

### EBV related miRNA regulation network

**Fig. 6:**
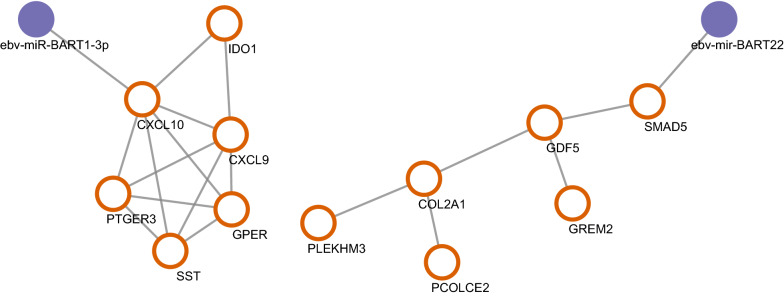
EBV related miRNA regulatory network (orange circles indicate target genes, purple dots indicate EBV related miRNAs)

Using ViRBase, we predicted the EBV related miRNA regulation network. After screening and matching, we found CXCL10 and SMAD5 were regulated by EBV related miRNA in the difference expression genes. CXCL10 was regulated by ebv-miR-BART1-3p, while SMAD5 was regulated by ebv-mir-BART22 (Fig. 6).

### Cross network

**Fig. 7:**
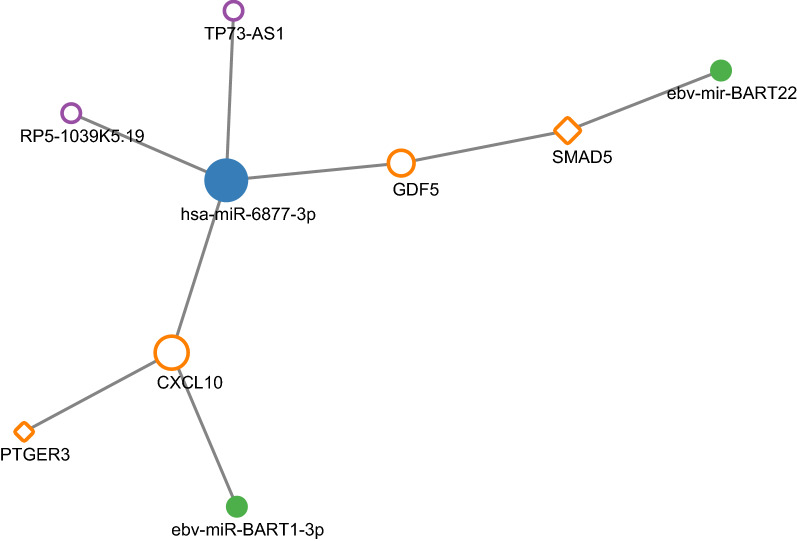
Cross network (orange circles indicate target genes, orange diamonds indicate transcription factors, blue dots indicate miRNAs, purple circles indicate lncRNAs, green dots indicate EBV related miRNAs, and size increases with degree)

Overlapping genes and regulators were observed in the cross network, including CXCL10, GDF5, PTGER3, SMAD5, miR-6877-3p, RP5-1039K5.19, TP73-AS1, EBV-miR-BART1-3p and EBV-mir-BART22 (Fig. 7). As for the two main target genes, compared with normal tissues, the expression of GDF5 was down-regulated while the CXCL10 was up-regulated significantly, with the logFC value of − 4.77 and 4.97, separately.

### Differential expressions of the target genes and transcription factors in the cross network in different molecular subtypes of GC

According to the mRNA expression data of different types of GC from cBioPortal, significant differences were observed between EBVaGC and EBVnGC for GDF5, CXCL10, SMAD5 and PTGER3 (P < 0.001, P < 0.001, P = 0.002 and P < 0.001). As for the four subtypes of GC, CXCL10 expression was significantly up-regulated in EBVaGC than in GS-GC, MSI-GC and CIN-GC (all P < 0.001). The expression levels of GDF5, SMAD5 and PTGER3 in EBVaGC were the lowest in the four subtypes (all P < 0.001). The significant differences were observed between EBVaGC and GS-GC for GDF5 (P < 0.001), and between EBVaGC and GS-GC/CIN-GC for SMAD5 (P < 0.001/P = 0.003) and PTGER3 (P < 0.001/P = 0.021) (Table 2).

**Table 2:** The mRNA expressions of target genes and TFs in the cross network in different molecular subtypes of GC by cBioPortal database

Gene	Tpye of GC	Sample number	mRNA expression	
			Mean ± SD	*P* value
GDF5	EBVaGC	29	2.11 ± 2.52	Ref
	EBVnGC	311	5.16 ± 9.55	< 0.001
				< 0.001*
	EBVaGC	29	2.11 ± 2.52	Ref
	GS-GC	47	10.46 ± 12.75	< 0.001
	MSI-GC	62	2.12 ± 2.68	0.995
	CIN-GC	202	4.85 ± 9.57	0.120
CXCL10	EBVaGC	30	6061.34 ± 6.56	Ref
	EBVnGC	346	759.53 ± 1.21	< 0.001
				< 0.001*
	EBVaGC	30	6061.34 ± 6.56	Ref
	GS-GC	50	352.97 ± 412.44	< 0.001
	MSI-GC	73	1523.12 ± 2.11	< 0.001
	CIN-GC	223	600.72 ± 743.04	< 0.001
SMAD5	EBVaGC	30	1165.21 ± 557.37	Ref
	EBVnGC	346	1446.01 ± 459.22	0.002
				< 0.001*
	EBVaGC	30	1165.21 ± 557.37	Ref
	GS-GC	50	1638.22 ± 483.08	< 0.001
	MSI-GC	73	1355.35 ± 330.26	0.058
	CIN-GC	223	1432.59 ± 479.20	0.003
PTGER3	EBVaGC	30	78.20 ± 91.63	Ref
	EBVnGC	346	191.35 ± 235.51	< 0.001
				< 0.001*
	EBVaGC	30	78.20 ± 91.63	Ref
	GS-GC	50	405.75 ± 309.97	< 0.001
	MSI-GC	73	101.47 ± 108.61	0.610
	CIN-GC	223	172.69 ± 218.64	0.021

* *P* value for overall comparison among four subgroups

### Verification of the target genes expression in the cross network

The results from human tissue verification of the two target genes showed that the gene expression level was lower in EBVaGC compared with that in non-tumor tissues for GDF5 (P = 0.043), and marginal difference was also observed between EBVaGC and EBVnGC (P = 0.076). As for CXCL10, its expression was higher in EBVaGC than that in control group with a borderline significance (P = 0.080). There is significant difference between EBVaGC and EBVnGC for CXCL10 (P = 0.047) (Table 3).

**Table 3:** Histological verification of the mRNA expressions of GDF5 and CXCL10

Gene	Group	mRNA expression	
		Mean ± SD	*P* value
GDF5	EBVaGC vs CON	0.025 ± 0.038 vs 0.254 ± 0.418	*0.043*
	EBVnGC vs CON	0.096 ± 0.096 vs 0.118 ± 0.199	0.893
	EBVaGC vs EBVnGC	0.025 ± 0.038 vs 0.096 ± 0.096	0.076
CXCL10	EBVaGC vs CON	0.867 ± 1.440 vs 0.205 ± 0.281	0.080
	EBVnGC vs CON	0.206 ± 0.138 vs 0.154 ± 0.164	0.686
	EBVaGC vs EBVnGC	0.867 ± 1.440 vs 0.206 ± 0.138	*0.047*

*CON* adjacent non-tumor tissue

## Discussion

The genetic and epigenetic regulation mechanisms can be clarified by examining mRNAs, TFs, miRNAs, lncRNAs and their networks. Our study conducted integrated analysis of gene regulatory networks based on TFs, miRNAs and lncRNAs targeting differentially expressed genes, and revealed key elements and their interactions associated with molecular mechanisms of EBVaGC.

Firstly, a total of 104 differentially expressed genes between EBvaGC and normal controls were identified from GEO databases using the GEO2R program in the present research. The functional analysis showed that these genes were mainly associated with digestion, G-protein coupled receptor binding, gastric acid secretion, etc. KEGG enrichment analysis also illustrated that the differential genes were mainly involved in the gastric acid secretion and protein digestion and absorption. Acid secretion exerts the greatest impact of all gastric functions on the occurrence of stomach disorders [28]. Our findings highlighted the probable importance of the regulation of these key genes and vital biological behaviors in EBVaGC, which warranted further investigations.

Furthermore, a set of gene regulatory networks were constructed by targeting these differentially expressed genes. At transcriptional level, studies have revealed that gene misregulation is often due to the aberrant expression of TFs. Based on the TF network, we identified some hub TFs associated with EBVaGC, including IRX3, NKX6-2, PTGER3 and SMAD5. Iroquois homeobox 3 (IRX3) plays vital roles in embryonic development, it has recently been reported to participate in tumor progression. Choi et al. [29] found that NKX6 participated in differentiation of gastrin-producing G cells in the stomach antrum. Prostaglandin E-receptor was observed to induce growth inhibition in gastric cancer cells [30]. Nagasako et al. [31] reported that up-regulated SMAD5 mediated apoptosis of gastric epithelial cells induced by Helicobacter pylori infection. These TFs may individually or comprehensively participate in EBVaGC pathogenesis by regulating their target genes, such as SST (Somatostatin) and GDF5 (growth differentiation factor 5). SST is important for regulating motor activity and the secretion of gastrin-stimulated gastric acid in the gastrointestinal tract [32], and GDF5 serves as a regulator of cell growth and differentiation in both embryonic and adult tissues. Their aberrant expressions were reported to be associated with varieties of cancers [33–36].

Noncoding RNAs (ncRNAs) are also important part of the regulatory network involved in post-transcriptional regulation of genes. By building ceRNA network, our results also revealed several novel miRNAs and lncRNAs that were possibly involved in gene regulation associated with EBVaGC. The top five miRNAs were hsa-miR-4446-3p, hsa-miR-5787, hsa-miR-1915-3p, hsa-miR-335-3p and hsa-miR-6877-3p. Kim et al. [37] observed that miR-4446-3p was upregulated by compression in breast cancer cells. Aberrantly expression of miR-5787 was supposed significantly down-regulated in serum and might be involved in the process of glucose metabolism in colorectal cancer [38]. miR-1915 inhibits Bcl-2 to modulate multidrug resistance by increasing drug-sensitivity of human colorectal cancer cells [39]. Overexpression of miR-335 significantly inhibited cell proliferation, migration and invasion in GC cells [40]. Little is known about miR-6877-3p, the only research reported that its expression was associated with ovary development in cyprinus carpio [41]. In addition, two unreported lncRNAs, RP5-1039K5.19 and TP73-AS1 were identified in the ceRNA regulation network, which may become the candidate targets for in-depth study of EBVaGC.

Additionally, miRNAs are not solely produced by metazoans, but also by viruses, which opened a new window for the research. Up to date, 44 mature EBV coding miRNAs have been identified, many of which have been proven to promote carcinogenesis by targeting host genes [13]. In our study, we built an EBV related miRNA regulation network and found that CXCL10 and SMAD5 were regulated by EBV-miR-BART1-3p and EBV-mir-BART22. EBV-miR-BART1 was observed to be involved in regulating metabolism-associated genes [42] and induced tumor metastasis [43] in nasopharyngeal carcinoma. Zhou et al. [44] found that CXCL10/CXCR3 axis can promote the invasion of GC via PI3 K/AKT pathway-dependent MMPs production. As for EBV-mir-BART22, it is a brand new miRNA without prior study. Interestingly, its target gene SMAD5 was also identified as a hub TF associated with EBVaGC in our study.

Intriguingly, when taking an overview on the various regulation networks in the current study, some overlapping genes and regulators were observed in the cross network. Firstly, CXCL10 was the common target gene in the three diverse regulation networks. It could be regulated by the transcription factor PTGER3, miR-6877-3p and EBV-miR-BART1-3p at the same time. Secondly, GDF5 was the target gene of transcription factor SMAD5 and miR-6877-3p. Moreover, SMAD5 was simultaneously regulated by EBV-mir-BART22. In addition, both CXCL10 and GDF5 were in the same ceRNA network that they can be regulated by miR-6877-3p and the two unreported lncRNAs, RP5-1039K5.19 and TP73-AS1. Furthermore, the expression levels of GDF5, CXCL10, SMAD5 and PTGER3 were also different between EBVaGC and EBVnGC. There were also differences between EBVaGC and other molecular subtypes of GC for these genes. In addition, in the histological verification experiment, differential expressions of the two main target genes GDF5 and CXCL10 were observed between EBVaGC and non-tumor tissues as well as EBVnGC. These results indicate that GDF5 and CXCL10 and their misregulation may play important roles specifically in EBVaGC related mechanisms. CXCL10 is a strong angiostatic factors, and it may be involved in the recruitment of tumour-infiltrating T cells [45]. It has been reported that TGF-β produced by breast cancer cells induces the GDF5 expression in the endothelial cells, which in its turn stimulates the angiogenesis both in vivo and in vitro [46]. Dysregulation of these two genes may lead to the activation of pathways related to cancer hallmarks like angiogenesis and tumour-promoting inflammation to promote EBVaGC, which needs further investigated. These identified key elements and their network regulation may offer new perspectives on mechanisms of EBVaGC.

## Conclusion

In summary, in current study, we provided a framework for revealing the key elements and their regulatory network involved in EBVaGC. Some hub TFs associated with EBVaGC, including IRX3, NKX6-2, PTGER3 and SMAD5 were found to regulate their target genes. We also identified five miRNAs hsa-miR-4446-3p, hsa-miR-5787, hsa-miR-1915-3p, hsa-miR-335-3p, hsa-miR-6877-3p and two unreported lncRNAs, RP5-1039K5.19 and TP73-AS1 in the ceRNA regulation network. EBV related miRNAs EBV-miR-BART1-3p and EBV-mir-BART22 were observed to regulate CXCL10 and SMAD5. Further, some overlapping genes and regulators were observed in the three diverse regulation networks, such as CXCL10, GDF5, PTGER3, SMAD5, miR-6877-3p, RP5-1039K5.19, TP73-AS1, EBV-miR-BART1-3p and EBV-mir-BART22. Moreover, CXCL10, GDF5, PTGER3 and SMAD5 were also differentially expressed among the four molecular subtypes of GC. The histological verification experiment showed differential expressions of the two main target genes GDF5 and CXCL10 between EBVaGC and non-tumor tissues as well as EBVnGC. Therefore, the misregulation of target genes GDF5 and CXCL10 may be specifically involved in EBVaGC mechanisms. This study provides a new insight into understanding the mechanism based on gene regulation of EBVaGC, and further molecular experiments are needed to confirm the findings.

## Additional file

**Additional file 1: Table S1.** The list of primers. **Table S2.** The list of identified differentially expressed genes.
