# Supplementary material for: Key elements involved in Epstein–Barr virus-associated gastric cancer and their network regulation
Source: Cancer Cell Int. 2018 Sep 21;18:146. doi: 10.1186/s12935-018-0637-5 (PMC6151003; doi:10.1186/s12935-018-0637-5)
Supplement: Supplementary file 1 — Additional file 1: Table S1. The list of primers. Table S2. The list of identified differentially expressed genes. [file 12935_2018_637_MOESM1_ESM.docx]

| Additional file: Table S1. The list of primers | |
| --- | --- |
| **Primer name** | **Sequence** |
| CSCL10-F | GTGGCATTCAAGGAGTACCTC |
| CXCL10-R | TGATGGCCTTCGATTCTGGATT |
| GDF5-F | AGACCGTGTATGAGTACCTGTT |
| GDF5-R | GTCCTTGAAGTTGACATGCAGT |

| Additional file: Table S2.The list of identified differentially expressed genes | | | |
| --- | --- | --- | --- |
| **GB_ACC** | **logFC** | **adj.P.Val** | **Gene symbol** |
| NM_001275 | -11.5 | 4.06E-15 | CHGA |
| NM_022469 | -5.2 | 4.06E-15 | GREM2 |
| NM_001079807 | -11.7 | 2.1E-14 | PGA3 |
| NM_152869 | -4.71 | 2.69E-14 | RGN |
| NM_000261 | -7.51 | 3.07E-14 | MYOC |
| NM_152459 | -5.92 | 3.08E-14 | C16ORF89 |
| NM_000704 | -11.3 | 3.59E-14 | ATP4A |
| NM_018476 | -4.23 | 3.98E-14 | BEX1 |
| NM_001005473 | -4.82 | 5.2E-14 | PLCXD3 |
| NM_003837 | -6.15 | 7.6E-14 | FBP2 |
| NM_032411 | -5.51 | 8.41E-14 | C2ORF40 |
| NM_001825 | -5.8 | 1.19E-13 | CKMT2 |
| NM_019617 | -10.4 | 2.32E-13 | GKN1 |
| NM_002371 | -4.52 | 2.95E-13 | MAL |
| NM_178125 | -7.97 | 4.42E-13 | TRIM50 |
| NM_001003395 | -5.05 | 4.53E-13 | TPD52L1 |
| NM_001144829 | -4.32 | 5.8E-13 | FMO5 |
| NM_015460 | -5.02 | 6.92E-13 | MYRIP |
| NM_000705 | -10 | 1E-12 | ATP4B |
| NM_198461 | -4.17 | 2.3E-12 | LONRF2 |
| NM_004469 | -4.02 | 6.19E-12 | VEGFD |
| NM_001565 | 4.97 | 6.74E-12 | CXCL10 |
| NM_006849 | -6.99 | 9.27E-12 | PDIA2 |
| NM_144575 | -4.12 | 1.93E-11 | CAPN13 |
| NM_175873 | -4.7 | 3.04E-11 | SOWAHA |
| NM_001080400 | -5.02 | 3.15E-11 | PLIN4 |
| NM_001048 | -6.31 | 3.71E-11 | SST |
| NM_016362 | -7.19 | 6.68E-11 | GHRL |
| NM_001869 | -5.55 | 7.69E-11 | CPA2 |
| NM_005142 | -9.01 | 7.69E-11 | GIF |
| NM_173833 | -4.53 | 8.91E-11 | SCARA5 |
| NM_001958 | -4.74 | 9.4E-11 | EEF1A2 |
| NM_172201 | -7.57 | 1.18E-10 | KCNE2 |
| NM_005060 | -4.3 | 1.33E-10 | RORC |
| NM_182983 | -5.2 | 1.82E-10 | HPN |
| NM_170741 | -5.64 | 2.56E-10 | KCNJ16 |
| NM_006789 | -5.1 | 2.59E-10 | APOBEC2 |
| NM_002416 | 4.41 | 3.42E-10 | CXCL9 |
| NM_017434 | -5.27 | 4.24E-10 | DUOX1 |
| NM_016725 | -5.09 | 4.98E-10 | FOLR1 |
| NM_005423 | -7.17 | 5E-10 | TFF2 |
| NM_001216 | -5.8 | 5.88E-10 | CA9 |
| NM_001166424 | -8.03 | 8.1E-10 | PGC |
| NM_001039966 | -5.63 | 9.65E-10 | GPER1 |
| NM_024336 | -4.39 | 9.72E-10 | IRX3 |
| NM_176875 | -5.57 | 9.79E-10 | CCKBR |
| NM_003167 | -4.38 | 1.07E-09 | SULT2A1 |
| NM_002164 | 4.53 | 1.19E-09 | IDO1 |
| NM_013363 | -4.1 | 1.75E-09 | PCOLCE2 |
| NM_000608 | -4.74 | 2.27E-09 | ORM2 |
| NM_001097620 | -8.81 | 2.34E-09 | TMEM184A |
| NM_002630 | -8.33 | 2.34E-09 | PGC |
| NM_005518 | -4.23 | 2.99E-09 | HMGCS2 |
| NM_058186 | -5.82 | 6.23E-09 | FAM3B |
| NM_033014 | -4.38 | 6.58E-09 | OGN |
| NM_003881 | -4.02 | 6.61E-09 | WISP2 |
| NM_181644 | -4.5 | 7.39E-09 | MFSD4A |
| NM_199330 | -4.21 | 9.49E-09 | HOMER2 |
| NM_005961 | -5.03 | 9.94E-09 | MUC6 |
| NM_001001419 | -4.64 | 1.11E-08 | SMAD5 |
| NM_004190 | -9.66 | 1.66E-08 | LIPF |
| NM_000371 | -4.74 | 1.92E-08 | TTR |
| NM_005950 | -4.81 | 2.35E-08 | MT1G |
| NM_001823 | -4.29 | 2.81E-08 | CKB |
| NM_177400 | -5.83 | 2.93E-08 | NKX6-2 |
| NM_004970 | -4.59 | 4.59E-08 | IGFALS |
| NM_005672 | -6.65 | 5.71E-08 | PSCA |
| NM_207581 | -6.16 | 5.96E-08 | DUOXA2 |
| NM_001824 | -4.96 | 6.81E-08 | CKM |
| NM_002407 | -4.79 | 0.00000007 | SCGB2A1 |
| NM_005951 | -4.15 | 7.21E-08 | MT1H |
| NM_002343 | -6.46 | 7.75E-08 | LTF |
| NM_001844 | -5.11 | 0.000000102 | COL2A1 |
| NM_004212 | -5.52 | 0.000000114 | SLC28A2 |
| NM_174924 | -5.22 | 0.000000167 | PDILT |
| NM_001085382 | -4.45 | 0.000000215 | PSAPL1 |
| NM_006103 | -4.39 | 0.000000269 | WFDC2 |
| NM_002112 | -4.15 | 0.000000273 | HDC |
| NM_031279 | -4.43 | 0.000000304 | ETNPPL |
| NM_139072 | -4.04 | 0.000000313 | DNER |
| NM_001010905 | -5.82 | 0.000000395 | C6ORF58 |
| NM_080870 | -5.41 | 0.000000528 | DPCR1 |
| NM_198719 | -4.18 | 0.000000615 | PTGER3 |
| NM_001650 | -4.82 | 0.000000688 | AQP4 |
| NM_001144950 | -6.19 | 0.000000753 | SSC5D |
| NM_000798 | -4.61 | 0.000000757 | DRD5 |
| NM_144947 | -4.91 | 0.00000105 | KLK11 |
| NM_001033113 | -4 | 0.00000121 | ENTPD8 |
| NM_054025 | -4.24 | 0.0000032 | B3GAT1 |
| NM_015464 | -4.52 | 0.00000362 | SOSTDC1 |
| NM_014080 | -5.36 | 0.00000363 | DUOX2 |
| NM_138938 | -5.6 | 0.00000414 | REG3A |
| NM_003225 | -4.36 | 0.00000575 | TFF1 |
| NM_053277 | -4.18 | 0.00000803 | CLIC6 |
| NM_000557 | -4.77 | 0.0000121 | GDF5 |
| NM_001008387 | -5.14 | 0.0000136 | REG3G |
| NM_007352 | -4.58 | 0.0000259 | CELA3B |
| NM_001080475 | -4.07 | 0.000036 | PLEKHM3 |
| NM_005747 | -4.11 | 0.000159 | CELA3A |
| NM_005073 | -4.43 | 0.000188 | SLC15A1 |
| NM_000039 | -4.23 | 0.000201 | APOA1 |
| NM_024339 | -4.29 | 0.000384 | THOC6 |
| NM_021797 | -5.79 | 0.000486 | CHIA |
| NM_006418 | 4.02 | 0.0091 | OLFM4 |
